# Review of acute kidney injury and progression to chronic kidney disease in pediatric patients undergoing hematopoietic cell transplant

**DOI:** 10.3389/fonc.2023.1161709

**Published:** 2023-05-23

**Authors:** Kim T. Vuong, Catherine Joseph, Joseph R. Angelo

**Affiliations:** Division of Pediatric Nephrology, Baylor College of Medicine and Texas Children’s Hospital, Houston, TX, United States

**Keywords:** hematopoietic cell transplant, chronic kidney disease, kidney injury, kidney transplant, nephrology

## Abstract

While acute kidney injury (AKI) after hematopoietic cell transplant (HCT) has been well-described in pediatric patients, literature regarding the long term renal consequences of HCT-related AKI, the development of chronic kidney disease (CKD), and CKD care in pediatric patients post-HCT is limited. CKD affects almost 50% of patients after HCT with multifactorial etiology including infection, nephrotoxic medications, transplant-associated thrombotic microangiopathy, graft-versus-host disease, and sinusoidal obstruction syndrome. As renal function declines in CKD, eventually progressing to end stage kidney disease (ESKD), mortality increases and is more than 80% among patients requiring dialysis. Using society guidelines and current literature, this review summarizes definitions and etiologies of and management strategies among patients with AKI and CKD post-HCT with an emphasis on albuminuria, hypertension, nutrition, metabolic acidosis, anemia, and mineral bone disease. The goal of this review is to aid early identification and intervention in patients with renal dysfunction prior to development of ESKD, and to discuss ESKD and renal transplant in these patients post-HCT.

## 1 Introduction

Hematopoietic cell transplant (HCT) is an established treatment for various malignant and non-malignant disorders among both adults and children. According to the Center for International Blood and Marrow Transplant Research, between 2008 and 2014, there were over 4400 pediatric allo-HCTs across 119 centers in the United States (1). Prevalence of acute kidney injury (AKI) after HCT has been reported as high as 70% in adult literature with variable incidence of 21% to 84% in pediatric literature (2, 3). In a previous review by Hingorani et al. in 2016, AKI within the first 30 days of transplantation and increase in AKI severity are associated with an increased risk of mortality, and mortality rates among those patients who require renal replacement therapy (RRT) ranges from 55-100%. A recent pediatric retrospective cohort study by Bauer et al. found that nephrology was consulted in less than 50% of patients with severe AKI, and risk of death was significantly higher in patients with severe AKI (RR 4.6, 95% CI 2.6-8.1) (4). Very little literature has been published about pediatric patients with chronic kidney disease (CKD) or who require RRT after HCT. The aim of this review is to describe the etiologies of kidney injury, strategies for AKI detection, and management of CKD in children following HCT. Given the broad scope of this review, we selected to focus on high yield topics and performed an unstructured search using PubMed to identify and summarize relevant literature.

## 2 Etiologies of kidney injury

**Figure 1:**
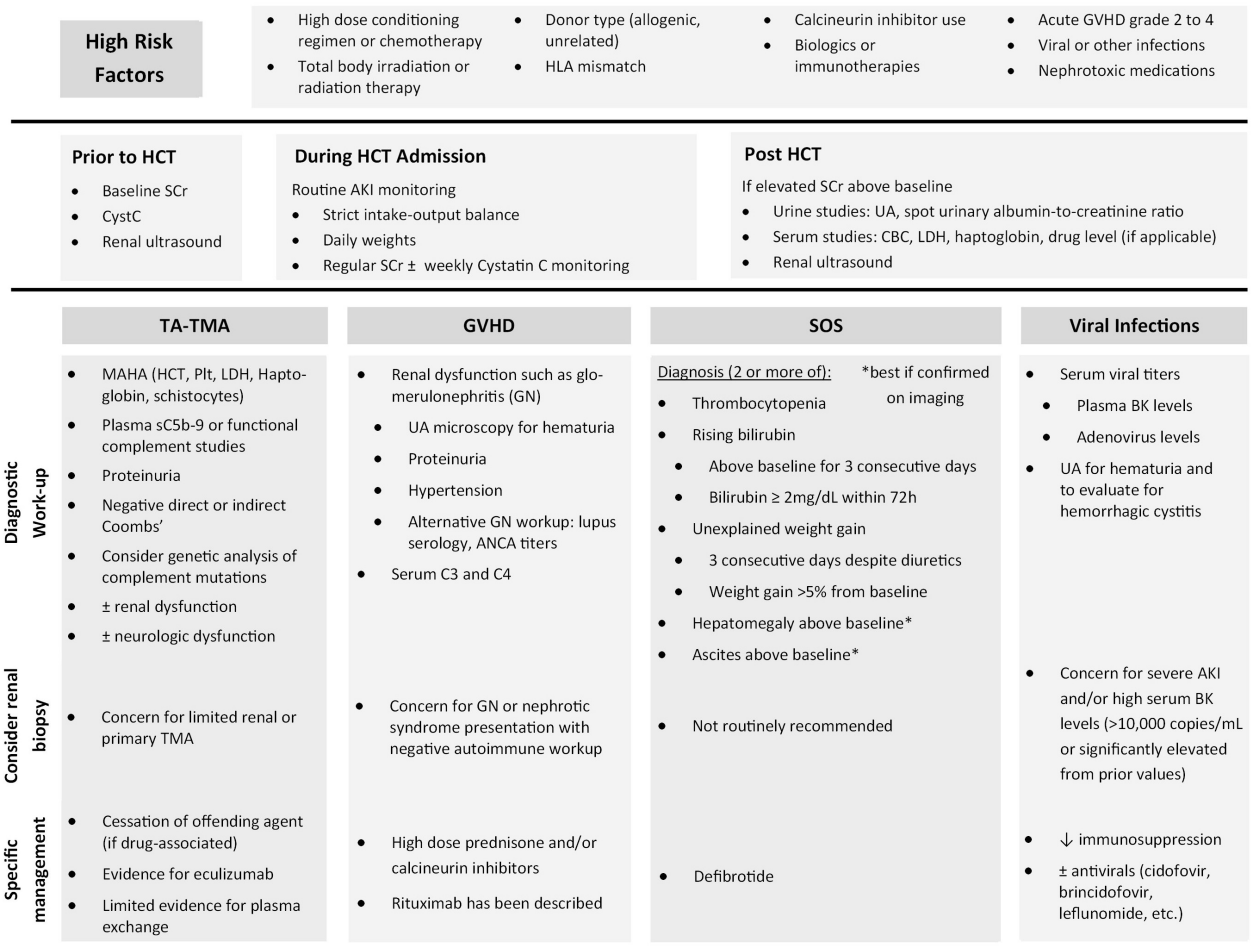
Summary of recommendations to identify high risk patients, etiology-based workup, and specific management.

Patients who undergo HCT have unique risk factors for AKI in addition to those of the general population. These can include nephrotoxic medication exposures such as antimicrobials, preconditioning chemotherapy, use of biologics or immunotherapies, radiation therapy possibly leading to radiation nephropathy, and use of calcineurin inhibitors (CNIs) as prophylaxis for graft-versus-host-disease (GVHD) (5). Mechanism of nephrotoxicity varies but has been categorized previously as vasoconstriction or altering intraglomerular hemodynamics, tubular cell toxicity, acute interstitial nephritis, tubular obstruction, hypersensitivity angiitis, and thrombotic microangiopathy (5). While CNIs can cause vasoconstriction of the renal artery, the exact mechanism between CNIs and AKI is unclear, as multiple studies have shown that neither the dose nor the drug level of CNIs in the blood, mostly cyclosporine, are significantly associated with AKI (2, 6). A recent review on CNI nephrotoxicity among renal transplant patients by Naesens et al. shows that local renal factors play a larger role than systemic overexposure to CNI, defined by CNI drug levels in the blood (7). These factors include patient variability in P-glycoprotein and CYP3A4/5 activity, older kidney transplant age, salt depletion and diuretic use, Non-Steroidal Anti-Inflammatory Drug (NSAID) use, and genetic polymorphisms in other genes such as ACE and TGF-β (7). Here, we discuss some unique clinical conditions that increase a patient’s risk of AKI after undergoing a HCT by focusing on the most prevalent etiologies and summarize our recommendations in **Figure 1**.

### 2.1 Transplant-associated thrombotic microangiopathy

Transplant-Associated Thrombotic Microangiopathy (TA-TMA) is characterized by endothelial dysfunction leading to microangiopathic hemolytic anemia (MAHA), thrombocytopenia, and multiorgan dysfunction typically within the kidneys, lungs, gastrointestinal (GI) tract, and central nervous system (8). Clinical diagnosis is made by the presence of MAHA, elevated lactate dehydrogenase level (LDH), renal dysfunction, and negative direct and indirect Coombs’ test. While some TMA diagnostic criteria, such as the Clinical Trials Network TMA criteria, include concurrent renal and/or neurologic involvement without another identified etiology, this criterion has been questioned for its validity and applicability to those patients with TMA at the highest risk of death (9, 10). Incidence is variable from 0.5-64% and likely impacted by variations in diagnostic criteria used across centers as well as differing pre-transplant conditioning regimens (11). In children and adolescents, those with TA-TMA had significantly greater non-relapse mortality at 1 year post-HCT compared to those without TA-TMA (43.6% vs 7.8%, P <0.0001) (12).

TA-TMA can be renally limited, but this does not always correlate with a rise in serum creatinine level. Kidney biopsy may be required to confirm the underlying diagnosis, especially in cases that exclusively involve the kidneys (13). A majority of TMA is caused by secondary TMA (80%-90%), and one of the most common forms is drug-induced TMA which makes up 10-13% of all TMAs and 20-30% of secondary TMAs (14, 15). Mazzierli et al. reviewed drug-induced TMA, which can present renally limited, and found complement deposition in half of the renal biopsies (57%, 37/66) of patients with complement deposition patterns associated with drug type, but also rare associations with pathological genetic mutations (1.6%, 2/122) (16). Similarly, abnormal complement activation without genetically identified complement disorders has been reported in a review of TMA caused by immune checkpoint inhibitors (17) and in an broad review of TMA among patients with C3 deposition on kidney biopsy (18).

Risk factors for TA-TMA include high dose conditioning regimen, high dose chemotherapy, total body irradiation, donor type (allogenic, unrelated), HLA mismatch, CNI use, acute GVHD grade 2 to 4, and viral or other infections (especially BK viremia) (2). High dose conditioning chemotherapy may cause direct endothelial injury while CNIs cause direct endothelial injury, increase thromboxane A2 and endothelin levels, and decrease nitric oxide and prostacyclin levels (19). Infections can cause endothelial injury directly or indirectly through inflammatory mediators such as TNF-alpha and IL-1 (11). GVHD is a risk factor for TA-TMA and is associated with a four-fold higher risk, independent of CNI levels or dosing regimen, and, conversely, an increased grade of GVHD leads to a higher risk of TA-TMA (13). While GVHD and TA-TMA can occur independently from one another, they can overlap clinically and both present with endothelial injury (20).

### 2.2 Graft-versus-host disease

GVHD is classically known to affect major organs including the skin, liver, and GI tract, but the kidney can also be involved. GVHD can present in the kidney as AKI, nephrotic syndrome, glomerulonephritis, or TA-TMA (8). Typical presentation is glomerulonephritis between 6 to 12 months after HCT, often as preventive immunosuppression in being weaned (21). While current GVHD grading does not include the kidneys, the presence of GVHD in other organs is a risk factor for kidney GVHD and is believed to be due to not only systemic inflammation but also local changes within the kidney, sharing similar GVHD-associated pathways as the other more common target organs (22). GVHD is independently associated with TA-TMA, likely due to targeting by donor graft cells to recipient’s vascular endothelium (13). The presence of GVHD is also an independent risk factor for AKI and CKD (23).

### 2.3 Sinusoidal obstruction syndrome

Sinusoidal Obstruction Syndrome (SOS), previously known as hepatic veno-occlusive disease, occurs after endothelial injury within hepatic sinusoids and hepatocytes in zone 3 of the hepatic acinus causes hepatocellular necrosis, fibrosis, and vascular occlusion. Left untreated, this can result in liver failure, hepatorenal syndrome, multiorgan failure, and eventually death (21). This recent meta-analysis by Raina et al. estimates a pediatric incidence of 18.2% (95% CI: 9.6-28.8%) compared to an older meta-analysis by Coppell et al. with almost 25,000 patients which found an overall mean incidence of 13.7% (95% CI: 13.3%-14.1%) and mortality rate from severe VOD of 84.3% (95% CI: 79.6-88.9%) (24). Risk factors for SOS include high dose conditioning regimens that lead to acute portal hypertension from direct injury to endothelial cells in the hepatic sinusoids and activation of stellate cells (2, 24). This leads to portal hypertension which can impact renal perfusion and cause renal tubular injury (2). SOS is an independent predictor of all stages of AKI, including severe AKI requiring RRT, and the presence of both SOS and AKI are associated with worse clinical outcomes (2, 3).

Diagnostic criteria for SOS in children by the European Society of Blood and Marrow Transplantation (EBMT) was first proposed in 2017 and includes 2 or more of the following: thrombocytopenia (unexplained, consumptive, transfusion-refractory), unexplained weight gain for 3 consecutive days despite diuretics or weight gain >5% from baseline, hepatomegaly above baseline (best if confirmed on imaging), ascites above baseline (best if confirmed on imaging), and rising bilirubin above baseline on 3 consecutive days or bilirubin ≥ 2mg/dL within 72h (25). EBMT pediatric severity grading for SOS had previously defined renal function scoring by glomerular filtration rate (GFR), but in 2019 the internationally accepted criteria for acute kidney injury (AKI) staging defined by Kidney Disease: Improving Global Outcomes (KDIGO) was used instead (26).

### 2.4 Viral infections

Viral infections can cause a variety of renal conditions. BK virus or adenovirus are common causes of hemorrhagic cystitis (HC). HC presents with hematuria, dysuria, flank pain, and potentially AKI. Risk factors for BK virus HC (BKV-HC) include treatment with rabbit thymocyte globulin, high BK virus levels, cord-blood or peripheral blood stem cell transplant, presence of GVHD (grade 2 to 4), age greater than 7 years old, and concurrent infection with other viruses. BKV-HC among pediatric patients is reported between 9.9% to 21.3% of patients after HCT (27, 28). Ruderfer et al. found that BKV-HC is associated with increased all-cause mortality (HR 2.22; 95% CI: 1.35-3.65), more severe AKI (stages 2 and 3) when occurring in the first 60 days post-HCT, and development of acute renal failure requiring dialysis and CKD stage 2-3 when occurring in the first year post-HCT.

Plasma BK virus levels are more indicative of renal involvement than urine BK virus levels. A prospective pediatric study by Cesaro et al. found plasma BK is predictive with a viral load of 10,000 copies/mL significantly associated with BK-HC in multivariate analysis (HR 6.1, P = 0.0006), and BKV-HC associated with significantly higher risk of mortality (HR 2.6, p = 0.018) (29). A retrospective study comparing outcomes between children based on degree of viremia found that those with plasma BK virus levels of at least 10,000 copies/mL during their first year post-transplantation had lower rates of survival at 1 year, worse renal disease (with 7/10 = 70% requiring dialysis), and more severe BKV-HC (including urologic complications requiring surgery) than children with levels less than 10,000 copies/mL (27). Patients with BK viremia and AKI should be considered for renal biopsy, if it can be safely obtained, to confirm diagnosis of BK virus nephropathy before treatment with antivirals such as cidofovir. Treatment includes reduction of immunosuppression, if possible, prior to initiating antivirals including leflunomide, cidofovir, and brincidofovir. Cidofovir has had mixed success for treatment of HC. Similarly, adenovirus has been identified as a cause of AKI post-HCT and is sensitive to both cidofovir and brincidofovir treatment (30).

## 3 Identification and monitoring for kidney injury in a patient with HCT

Kidney injury associated with HCT has a prevalence of 10-70% in adult literature with median time to onset of AKI 33 to 38 days after transplantation (2). In pediatric literature, AKI incidence varies widely from 21 to 84%, likely due to variability in AKI definitions and patient heterogeneity (3). Consensus statement by the pediatric continuous renal replacement therapy (PCRRT) working group meta-analysis showed statistically significant higher AKI rate among allogenic (39.3%, 95% CI: 25.7–53%) than autologous transplant recipients (5%, 95% CI: 0–11.9%) as well as higher AKI incidence in patients with HCT due to malignancy (33.6%) than those undergoing HCT without malignancy (6.1%) (21). Those patients who have significant risk factors for AKI benefit from close monitoring to allow for early detection of AKI which can be challenging in pediatric patients.

### 3.1 Diagnosing AKI

There are several criteria published in the literature to diagnose AKI. These include the risk, injury, failure, loss of kidney function, and end stage kidney disease (RIFLE) system, the acute kidney injury network (AKIN) criteria for kidney injury, and KDIGO criteria (2, 31). As it is currently the most widely used and internationally accepted system for staging AKI, we recommend the KDIGO system (shown below in **Table 1**) for pediatric patients.

**Table 1:** KDIGO staging of AKI based on serum creatinine and urine output (31).

Stage	Serum Creatinine (SCr)	Urine output
**1**	1.5-1.9x baseline Scr ≥ 0.3mg/dL above baseline	< 0.5ml/kg/hr for 6-12h
**2**	2-2.9x	< 0.5ml/kg/hr for ≥ 12h
**3**	3x baseline SCr SCr ≥ 4mg/dL Initiation of renal replacement therapy	< 0.3ml/kg/hr for ≥ 24h, or Anuria for ≥ 12h

While current KDIGO criteria relies on SCr and increasing duration of oliguria to detect AKI, SCr can be a suboptimal AKI biomarker. SCr indicates actual loss of kidney function from an injury that occurred 48-72 hours prior and overestimates GFR due to tubular secretion. SCr is also impacted by sex, age, height, protein intake, and muscle mass (31–33). The most studied alternative AKI biomarker for pediatric patients is Cystatin C (CysC), a 13 kDa cysteine protease inhibitor that is freely filtered by the glomerulus and without any known tubular secretion (32). As a functional biomarker, serum CysC reflects a change in kidney function, rather than a loss of function, and can detect AKI earlier than SCr. However, some studies show that serum CysC can be influenced by inflammation, steroids, and age (32, 34–39). A systematic review and meta-analysis by Zhang et al. of primarily adult studies showed serum CysC was able to predict AKI with a diagnostic odds ratio (OR) of 23.5 (95% CI: 14.2-38.9), sensitivity of 0.84, specificity of 0.82, and area under the receiver operating characteristics curve of 0.96 (95% CI: 0.95-0.97). GFR estimating equations based solely on CysC or incorporating CysC and SCr exist for children. The three most common equations for estimating GFR include the bedside Schwartz equation based on Creatinine (40), Cystatin-C based equation (41), Creatinine-Cystatin C-based Chronic Kidney Disease in Children (CKiD) equation (42), and U25 modification for CKiD equation for patients under 25 years old (43).

Alternative biomarkers have been studied for more accurate diagnosis, but many are not currently available for widespread use. These include tubular injury biomarkers like urinary Neutrophil gelatinase-associated lipocalin (NGAL), urinary N-acetyl-beta-D-glycosaminidase (NAG), kidney injury molecule-1 (KIM-1), tissue inhibitor of metalloproteinases-2 (TIMP-2) and insulin-like growth factor binding protein 7 (IGFBP-7). Data also exists for urine CXCL10 and CXCL9 in identifying AKI after HCT (44). Biomarkers of glomerular function and tubular injury can be combined with traditional markers (serum creatinine) for early AKI identification, especially within 28 days post-HCT. Benoit et al. monitored for AKI with weekly creatinine, cystatin C, and urinary NGAL to help identify highest risk patients for adverse outcomes (45).

### 3.2 Screening and evaluation for AKI

Prior to HCT, renal function should be evaluated with baseline SCr and CysC as well as imaging to evaluate for structural renal abnormalities with ultrasound. Having a pre-HCT baseline SCr and CysC allow for more accurate staging and recognition of AKI post-HCT. During initial HCT admission, we recommend routine application of KDIGO AKI criteria to identify patients during earlier stages of AKI when intervention may prevent requirement prolonged AKI, severe AKI, or requiring renal replacement.

Those patients who are found to have an elevated creatinine post-HCT compared to their pre-HCT baseline should undergo further investigation to determine the cause, typically with a combination of urine studies (complete urinalysis, spot urinary albumin-to-creatinine ratio, urine sodium, urine urea, and urine creatinine), serum studies (complete blood count, serum lactate dehydrogenase (LDH), haptoglobin, and drug levels of calcineurin inhibitors, if applicable), serum viral studies for BK virus and adenovirus DNA, renal ultrasound (to assess for kidney size and look for signs of obstructive uropathy), and, if diagnosis is still unclear, possibly kidney biopsy (2, 19). If there is concern for TA-TMA, recommended evaluation includes hematocrit, platelet, LDH, haptoglobin, peripheral smear for schistocytes, urine studies for proteinuria, and plasma sC5b-9 level. If glomerulonephritis from GVHD is suspected, evaluation should include urinalysis with microscopy, serum complement C3 and C4, lupus serology, ANCA titers, and possible kidney biopsy (21).

## 4 Management of AKI in a patient with HCT

KDIGO practice guidelines recommend management of AKI based on stage of AKI. For those patients at risk of AKI, nephrotoxic agents should be discontinued where clinically possible, effective circulating volume should be optimized to ensure adequate perfusion pressure, functional hemodynamic monitoring should be considered, serum creatinine and urine output should be monitored closely, hyperglycemia should be avoided due to risk of osmotic diuresis, and radiocontrast should be avoided when clinically possible. Diagnostic workup should be considered starting at stage 1 AKI with possible changes in drug dosing by stage 2-3 and consideration for ICU admission and/or renal replacement therapy (RRT) (31).

### 4.1 Fluid balance

Among children with AKI after HCT, increased fluid overload is independently associated with worse clinical outcomes and increased mortality (46). Based on a review by Raina et al, fluid overload more than 10-20% is also associated with increased mortality, ICU length of stay, and mechanical ventilation (47). Due to this, these patients should be monitored closely for fluid overload including strict fluid intake and output, surveillance of percent fluid overload as part of their daily assessment, and targeted net fluid balance goals per day (21). To achieve these fluid balance goals, a study by Raina et al. recommended an algorithm to monitor daily fluid status after HCT and consider initiation of furosemide infusion for fluid overload of at least 5% (48). In the setting of increasing weight and decreased urine output, consultation with nephrology and restriction of fluid volume is recommended due to the possible need for RRT.

### 4.2 Renal replacement therapy

Several adult, and combined adult and pediatric studies, have shown that the degree of renal failure is associated with mortality, and mortality can be as high as 84% (49, 50). A meta-analysis by Raina et al. of pediatric patients post-HCT found that 31.1% (95% CI: 17.1-47.2%, P<0.0001) of patients with AKI required RRT, most often due obligatory fluid requirements significantly exceeding urine output (21). These investigators also found that indicators of successful termination of continuous RRT (CRRT) include improvement in fluid overload state and urine output irrespective of the use of diuretics at the time of discontinuation of CRRT (51). To help identify high risk characteristics of those patients who required RRT, a retrospective study by Lane et al. compared 30 pediatric patients who required dialysis early after HCT to general pediatric HCT patients. Compared to general HCT pediatric patients, those requiring dialysis had a greater proportion of neuroblastoma as well as fewer autologous and more unrelated HCT donors, potentially related to differences in conditioning regimen, GVHD prophylaxis, and infection prophylaxis or complications. While 77% (23/30) died without renal recovery mostly from sepsis, 23% (7/30) had renal recovery and survived. Clinical factors associated with persistent renal failure included requiring at least 3 medications for blood pressure support, hyperbilirubinemia, and fluid overload by weight >10% at RRT start (52). Reduced renal reserve among these patients was believed to be due to prior chemotherapy, tumor debulking surgery sometimes requiring nephrectomy, or prior abdominal radiation (53).

### 4.3 Etiology-based management

Targeted interventions focused on suspected etiology of AKI should also be performed. Nephrotoxic agents should be minimized or avoided as clinically able. For clinically necessary but potentially nephrotoxic medications, drug levels should be monitored closely with dosing adjusted based on estimated renal function. Glomerulonephritis due to GVHD can be treated with high dose prednisone and/or calcineurin inhibitors. Rituximab use has been described as well (54). For SOS, rapid and definitive diagnosis as well as timely initiation of defibrotide is critical. Defibrotide is a polydeoxyribonucleotide with aptameric activity on the endothelium and anti-inflammatory, anti-thrombotic, and anti-ischemic effects to counter the endothelial cell damage. Defibrotide is the only drug approved for prevention and treatment of SOS in the United States since it has been shown in a randomized controlled trial to reduce incidence of SOS-associated kidney failure (55).

For TA-TMA, especially secondary or drug-induced, management should focus on withdrawal of the inciting agents such as calcineurin inhibitors, antimicrobial treatment for any active infection, and maintenance of metabolic balance by correcting fluid overload. There is limited evidence for the use of plasma exchange which has unclear benefit (lacks randomized controlled trial but anecdotal studies show <50% response and mortality >80%) while Eculizumab has been shown to improve outcomes in severe TA-TMA with 1 year survival and case reports supporting its use (56). However, it should be noted that several reviews report conflicting efficacy with therapeutic complement inhibition such as Eculizumab and low rates of pathogenic variants in complement genes in patients with TMA, especially drug-induced TMA. These authors caution that improvement after Eculizumab may be impacted by natural disease course after withdrawal of the offending agent in drug-induced TMA (16, 18).

### 4.4 AKI leading to CKD

Early nephrology involvement is recommended given even mild AKI can be associated with residual kidney damage. If etiology is unknown, biopsy should be considered if there is significant or persistent AKI after HCT. Biopsy could provide evidence of kidney involvement by GVHD, TA-TMA, or viral infections, allowing for more targeted treatment. Given most processes in the kidney leading to AKI are inflammatory, early identification and targeted treatment can help reduce duration of inflammation, reducing the progression to fibrosis and eventual irreversible kidney damage with loss of renal tubules and glomerular function (8). Recurrent renal GVHD can lead to tubular atrophy, peritubular capillary loss, and interstitial fibrosis. Recurrent episodes of AKI also increase the risk of progression to CKD. AKI is indicated by elevated serum Cr up to 100 days after HCT, chronic injury at or after 100 days, and CKD if AKI persists for 3 months or longer (2). After HCT, recommended evaluation for persistent kidney injury should occur at the 6- and 12-month post-transplant evaluation followed by at least yearly evaluations of serum blood urea nitrogen (BUN), serum creatinine, urinalysis, and blood pressure (21).

## 5 Management of CKD in a patient with HCT

It is important to identify those patients who have progressed from AKI to CKD due to the long term health implications and complications of CKD that require closer monitoring. Krist-van Holthe et al. found that high SCr within 3 months of HCT correlated with CKD at 1 year after HCT, so it is important to follow high risk patients long-term who have had prior AKI (57).

### 5.1 Definition

The National Kidney Foundation Kidney Disease Outcome Quality Initiative (KDOQI) consensus guidelines from 2012 define CKD as at least 3 months of either decreased GFR (GFR < 60ml/min/1.73m^2^) or one or more markers of kidney damage (albuminuria of at least 30mg/g, urine sediment abnormalities, electrolyte and other abnormalities due to tubular disorders, abnormalities detected by histology, structural abnormalities detected by imaging, or history of kidney transplantation) (58). It should be noted that while these guidelines do not include patients with stage 2 CKD (defined by GFR 60-89 ml/min/1.73m^2^), we recommend a similar monitoring and management strategies for these patients as they are still at risk of progressive renal dysfunction.

### 5.2 Etiology and risk factors

CKD is common among pediatric patients post-HCT with an incidence of 48% between 6mo and 10yr following HCT and with typical etiologies being idiopathic, TA-TMA, nephrotic syndrome, AKI, and drug toxicity usually attributed to calcineurin inhibitors (54). CKD caused by total body irradiation (TBI) or TA-TMA typically present 6-12mo following HCT (59). Risk factors include baseline GFR below 90ml/min/1.73m2, TBI exposure, nephrotoxic medications especially calcineurin inhibitors, infections including sepsis, recurrent AKI episodes, hypertension, and GVHD especially chronic GVHD due to prolonged immune-mediated renal damage (19).

### 5.3 CKD staging

As shown in **Table 2**, KDOQI and KDIGO recommend staging CKD by a combination of GFR as well as degree of albuminuria due to adult evidence that both are independently related to increased mortality, rates of end stage kidney disease (ESKD) progression, and cardiovascular events (58). Based on rate of CKD progression, acute medical events, or AKI episode, labs for CKD progression should be monitored closely. During acute complications or AKI, we recommend weekly CysC and at least weekly SCr. Outside of an acute episode, minimum frequency of monitoring for proteinuria/albuminuria, CysC, and SCr should occur with frequency based on CKD stage and degree of albuminuria (frequency of lab monitoring based on CKD stage is summarized in **Table 3**).

**Table 2:** Staging of CKD by GFR and degree of albuminuria from KDOQI US Commentary on the 2012 KDIGO Clinical Practice Guideline for the Evaluation and Management of CKD (58).

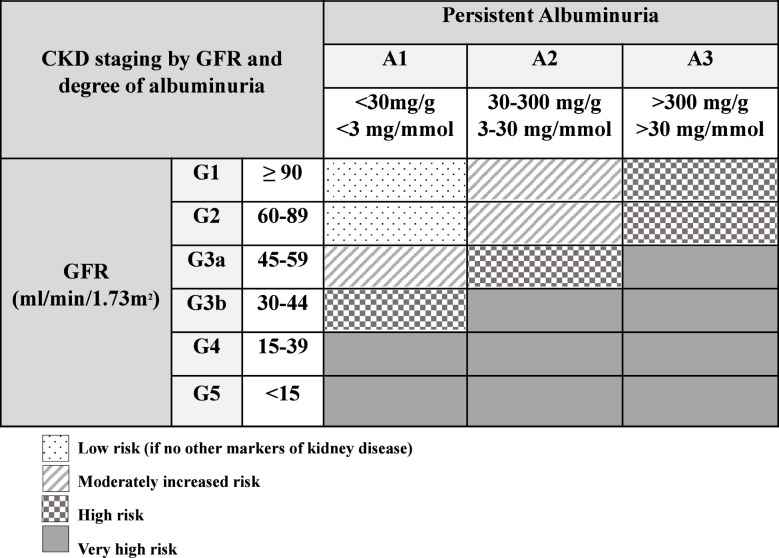		

**Table 3:** Frequency of monitoring based on CKD stage.

CKD Stage	Frequency of Lab Monitoring based on CKD stage											
	CKD Progression		Blood Pressure		Growth Monitoring		Anemia		CKD-MBD			
	CysC, SCr	Albuminuria	No HTN	HTN	Infants	Children	No anemia	Anemia	Ca, Phos	PTH	Vitamin 25(OH)D	AlkPhos
1	12mo	6*-12mo	In-office (12mo)	In-office (every visit) ABPM (12mo) Home BPs	Weight, Linear, HC (12mo)	Weight, Linear, +/- HC (12mo)	12mo	6mo	12mo	12mo	12mo	–
2	12mo	6*-12mo	In-office (3-6mo) ABPM (12mo)		Linear (3mo) Weight (6mo) HC (6mo) Dietitian	Linear (12mo) Weight (6mo) Dietitian	12mo	6mo	12mo	12mo	12mo	–
3a	12mo	4*-12mo					12mo	3mo	6-12mo	Baseline and based on CKD progression	Baseline and 3-6mo based on level and supplements	–
3b	6mo	4*-6mo					12mo	3mo	6-12mo			–
4	4mo	1*-4mo					6mo	3mo	3-6mo	6-12mo		–
5	1-3mo	1*-3m					3mo	1mo	1-3mo	3-6mo		12mo dependent on PTH

Asterix (*) indicates more frequent monitoring if the patient has significant albuminuria (A2 or A3).

### 5.4 General management

Similar to AKI management, it is important to slow progression of CKD by minimizing nephrotoxic exposures and AKI episodes, dosing medications based on estimated GFR, measuring drug levels when available, ensuring adequate hydration, optimizing blood pressure, and minimizing proteinuria. If hypertension and proteinuria are identified, initiation of an angiotensin converting enzyme inhibitor (ACE-I) or angiotensin receptor blocker (ARB) is recommended as this has been shown to be renoprotective long term at slowing progression of CKD, especially among those patients with significant proteinuria (19). All patients who meet criteria for CKD should be referred for nephrology consultation. Close follow-up with a nephrologist is critical to monitor regular labs for CKD complications including, but not limited to, abnormal blood pressure, nutrition issues, acidosis, anemia, and mineral bone disease. Multidisciplinary collaboration should include a dietitian who ideally has expertise in pediatric and renal nutrition, especially as patients without a gastrostomy tube may face challenges with oral tolerance during episodes of mucositis or esophagitis. Children with CKD benefit from multidisciplinary care given their risk of not only post-HCT complications but also CKD complications.

### 5.5 Complications of CKD: albuminuria

Albuminuria is associated with progression of CKD as well as decreased post-transplant survival (60). Development of any albuminuria in the first 100 days after HCT has been associated with increased risk of death at 1 year post-transplant (61). The most accurate method for assessing urinary albumin is a 24 hour urine collection. However, this can be difficult to accurately obtain particularly in younger patients. In a review by Hingorani et al, urinary albumin-to-creatinine ratios measured at days 80 to 100 days after transplantation has been shown to be predictive of subsequent kidney function and death, so any albuminuria during this time frame is associated with an increased risk of progression to CKD (2).

Albuminuria after HCT can occur as early as 2mo post-transplant, but typically occurs 6-12mo post-transplant which is temporally associated with discontinuation of immunosuppressive GVHD prophylaxis. Typical presentations include glomerulonephritis or nephrotic syndrome (NS). NS presents as proteinuria, hypoalbuminemia, edema, and hypercholesterolemia. NS in a patient who has undergone HCT should be evaluated with a renal biopsy as 60-80% of cases are due to membranous nephropathy, followed by 22% with minimal change disease (62). NS usually resolves after high dose prednisone treatment, restarting calcineurin inhibitors, or both. Rituximab has also been used successfully, typically among patients with membranous nephropathy after HCT.

If macroalbuminuria (>300mg albumin/g Cr) is detected, then the patient should be started on an ACE-I or ARB, and albuminuria should be monitored every 3-6 months. Macroalbuminuria can be a marker of renal GVHD, so it may be useful to continue immunosuppression after 100 days after transplantation even if there is no other evidence of GVHD in other organ systems (2). Macroalbuminuria as well as hypertension may also be early markers of TA-TMA in patients who have undergone HCT (12). If microalbuminuria (30-299 mg albumin/g Cr) is detected, then this should be repeated at least twice in the following 3-6 months to ensure albuminuria is stable before spacing monitoring to every 3-6 months. If the patient has microalbuminuria and hypertension then they should also be started on an ACE-I or ARB (63).

### 5.6 Complications of CKD: hypertension and blood pressure

Presence of HTN after HCT has been associated with poor long-term outcomes including increased risk of death, increased risk of CKD (in adult patients OR 4.03; 95% CI: 1.04-13.06 (64)), and increased risk of TA-TMA (19). HTN is defined as blood pressure greater than the 95^th^ percentile for sex, age, and height as measured on at least 3 occasions. HTN incidence has been reported as high as 80% in the first 2 years post-HCT in pediatric patients, typically occurring 1 month after HCT (65). A long term retrospective study by Hoffmeister et al. analyzing long term survivors of pediatric HCT found a 30-year cumulative incidence of 36% with median follow-up 16 years (range 5-36 years) and prevalence of 15% overall, which is 2-3 times that of the general population. Risk factors associated with HTN included AKI defined as doubling of baseline creatinine by day 100 after HCT, total body irradiation in preparative regimen, autologous donor type more so than unrelated donor type, obesity, diabetes, and history of growth hormone therapy. Longitudinal observation studies have shown that younger age, higher body mass index, and higher proteinuria, especially nephrotic range proteinuria, is more strongly associated with increasing BP over time (66).

BP should be checked using validated pediatric equipment with correct cuff size and position on upper extremity, as able. Ideally, patients should be seated with their feet on the floor and with arms and back supported. Patients should be relaxed and not talking or moving for at least 5 minutes prior to BP being measured (67). For young patients manual BP may be necessary due to intolerance. Standardized in-office blood pressure should be taken at least every 3-6 months. Where available, 24 hour mean arterial pressure by ambulatory blood pressure monitor (ABPM) should be performed at 80 days after transplantation and then annually, as this can provide a more accurate assessment of blood pressure variability and can help evaluate for white coat and masked HTN.

Treatment of HTN can help decrease not only progression of CKD but also cardiovascular disease risk. Lifestyle modifications should be implemented followed by antihypertensive treatment when BP is consistently >90^th^ percentile for age, sex, and height. First line chronic antihypertensives in CKD are typically renin-angiotensin-aldosterone system antagonists (e.g. ACE-I and ARBs), and calcium channel blockers are also commonly used. Patients should be provided medical grade BP monitor for home and instructed to track daily BP log with notification parameters for high or low BP values. After BP monitor is obtained, it should be brought to clinic to confirm correlation with in-office BP monitor. If patient does not have electrolyte derangements requiring sodium supplementation, sodium restriction to <2g/day or adjusted for body size based on dietitian evaluation is also recommended (67, 68).

Among patients with CKD, target BP goals should be less than or equal to 50^th^ percentile for age, sex, and height unless achieving this is limited by symptoms or signs of hypotension (67). This recommendation is based on the largest randomized control trial in pediatric CKD patients studying BP targets: the Effect of Strict Blood Pressure Control and ACE Inhibition on the Progression of CKD in Pediatric Patients (ESCAPE) trial. The ESCAPE trial included 385 children with baseline CKD with eGFR 20–80 ml/min/1.73m2 and 24 hour average ambulatory MAP >95th percentile. Children were randomized to intensified BP control (24 hour MAP <50^th^ percentile) or to standard BP control (24 hour MAP 50^th^-99^th^ percentile) using ramipril. The primary composite endpoint of 50% GFR decline and ESKD favored the intensive BP arm (HR: 0.65; 95% CI: 0.44–0.94) (69).

Children with systemic HTN have a narrower range of autoregulation in cerebral blood flow and an increased risk of cerebrovascular dysfunction, making them more susceptible to posterior reversible encephalopathy syndrome (PRES) (70). PRES presents with an abrupt, acute rise in BP along with seizures, visual changes, encephalopathy, headache, and radiologic findings on brain magnetic resonance imaging (MRI) with focal reversible vasogenic edema (71). PRES has been associated with a significant increase in length of stay and, when severe or if there are delays in treatment, can lead to secondary complications including ischemic infarction, intracranial hemorrhage, and status epilepticus. PRES is a neurological emergency requiring early identification and management including gradual reduction in BP and removal of any identifiable offending agents (72). Shah et al. studied the difference between PRES in pediatric oncology and post-HCT patients and found that oncology patients developed PRES at a younger age and were more likely to present with encephalopathy (70). Systemic HTN preceded PRES in 43.5% of patients, and this was more likely in post-HCT patients. Post-HCT patients were more likely to have rare neurological clinical presentations and more likely to die due to PRES-related complications.

### 5.7 Complications of CKD: nutrition and acidosis

Patients with CKD are at increased risk of protein energy malnutrition which can significantly impact linear growth, neurocognitive development, and sexual development (73). For patients with CKD stage 2-5, we recommend patients are seen by a dietitian who ideally has expertise in pediatric and renal nutrition. This allows patients to receive targeted recommendations and education based on their severity of CKD and any necessary dietary modifications including fluid, sodium (especially if hypertensive), phosphate, potassium, and protein goals. We do not recommend restricting nutrition without specific guidance from a renal dietitian given the patient’s increased risk of protein energy malnutrition.

Growth parameters should be evaluated at least twice per year with greater frequency among patients with acute medical issues, polyuria, concern for growth delay, declining or low BMI, or decreased nutritional intake (73). Infants with CKD stage 2-5 should have length measured at least every 3 months, and children with CKD stage 2-5 should have linear growth measured at least annually (74). According to the National Kidney Foundation’s recommendations of 2009, anthropometric parameters should include percentiles and standard deviation score (SDS) for length-for-age or height-for-age, length or height velocity for age, weight-for-age, BMI-for-height-age, head circumference-for-age (if less than 3 years of age). Patients with CKD after HCT are at higher risk for long term growth issues given frequent mucositis or GI symptoms limiting consistent nutrition delivery. A long term pediatric study by Perkins et al. of patients who received HCT under 3 years of age for acute lymphoblastic leukemia or acute myelogenous leukemia found growth hormone deficiency in 59%, abnormal pubertal development in 12%, and dyslipidemias in 59% (75).

Persistent metabolic acidosis can contribute to short stature and decreased linear growth potential, so patients with CKD stage 2-5 should have serum bicarbonate corrected to at least 22 mm/L (the lower limit of normal), often times requiring bicarbonate supplementation to achieve these goals. Typically in conjunction with endocrinology, initiation of recombinant human growth hormone (rhGH) therapy can be considered in patients with short stature (height SDS < 1.88 or height for age <3%ile) or linear growth failure (height velocity for age SDS < -1.88 or height velocity for age <3%ile) persisting for at least 3 months despite correction of metabolic derangements and nutritional deficiency. Contraindications to rhGH include pre-existing intracranial hypertension that could be worsened by rhGH, closed epiphyses, severe secondary hyperparathyroidism (PTH > 500pg/mL), proliferative or non-proliferative diabetic retinopathy, active malignancy, acute critical illness, within 1 year after renal transplantation, or known hypersensitivity to any component of rhGH medication (76).

### 5.8 Complications of CKD: anemia

As renal function declines, the kidneys are unable to synthesize adequate levels of erythropoietin which can lead to a progressively more severe anemia. Anemia is associated with increased mortality and hospitalization frequency in both adults and children. Left untreated, anemia can lead to cardiovascular dysfunction, decreased quality of life, impaired cognition, and reduced exercise capacity (77). Hemoglobin (Hgb) decline is gradual over time among patients with CKD but becomes a linear relationship with GFR below 43 ml/min/1.73m2, according to the CKiD Prospective Cohort Study which analyzed 340 North American children with CKD (78).

The diagnosis of anemia in children with CKD varies depending on age. Regardless of age or CKD stage, the evaluation for anemia should include: complete blood count (including Hgb concentration, red cell indices, white blood cell count, differential, platelet count), absolute reticulocyte count, serum ferritin level, serum transferrin saturation (TSAT), serum vitamin B12 and folate levels (78). After initial evaluation, anemia evaluation frequency with Hgb is based on CKD staging as shown in **Table 3**. All CKD patients with anemia should be started on enteral iron if their TSAT ≤ 20% and ferritin ≤ 100ng/mL. For patients with CKD 5, erythropoiesis stimulating agents (ESA) are considered but should target lower Hgb range of 10.0 to 12.0 g/dL among patients with cancer (79). During ESA therapy, iron status (TSAT and ferritin) should be evaluated at least every 3 months. Patients should be counseled about and receive routine monitoring for possible adverse effects such as thromboembolic events, hypertension, thrombocytopenia, seizure, or hemorrhage, skin issues such as rash, irritation, or pruritus (80).

Limited pediatric randomized control trials (RCTs) show improved hemoglobin level, decreased transfusion need, and a positive correlation between hemoglobin changes and health related quality of life changes among those who received ESAs, but these RCTs had short treatment and follow-up periods (8 weeks (81), 16 weeks (80), 12 weeks of treatment then follow-up for 7 years (82)). A Cochrane review including one pediatric RCT (80) found ESA use reduced the risk of red blood cell transfusion by an average of one unit of blood (RR 0.65, 95% CI 0.62-0.68, 70 trials, N=16,093) (83).

While patients with CKD after HCT are at a higher risk for anemia given factors associated with underlying etiology requiring HCT, frequent infections and blood draws, and medication side effects, the use of ESAs in patients with cancer is controversial due to concern that ESAs may directly stimulate tumor growth and lead to worse outcomes. There is strong evidence between ESA use and increased mortality during active study period (HR 1.17, 95% CI 1.06 to 1.29, 70 trials, N=15,935), increased risk of thromboembolic complications (RR 1.52, 95% CI 1.34 to 1.74; 57 trials, N=15,498), suggestive but not robust evidence of increased risk of hypertension (RR 1.24, 95% CI 1.09 to 1.58), but insufficient evidence about ESA effect on tumor response (fixed effect RR 1.02, 95% CI 0.98 to 1.06, 15 trials, N=5,012) (83). A large (n=13,933) meta-analysis of primarily adult clinical trials (<1% in ESA group were < 18 years of age) found similar results (84). Due to these potential risks, clinical practice guidelines recommend against systematic administration of ESAs in children with oncologic diseases and a case-by-case decision by renal and oncology teams for those patients with barriers to transfusions. Lower hemoglobin targets of 10 g/dl have been proposed to minimize risk of thrombosis and mortality (79, 85).

### 5.9 Mineral bone disease (CKD-MBD)

Based on the National Kidney Foundation KDIGO work group, patients with CKD, especially stage G3a-G5D, have significantly higher fracture rates than the general population, and infants and children with CKD suffer growth retardation and severe short stature (73, 74). Patients with CKD after HCT are at an increased risk for MBD given prolonged exposure to medications that affect bone metabolism such as systemic steroids. The pediatric study by Perkins et al. found patients who received HCT under 3 years of age had decreased bone mineral density in 24% and short stature in 47% (75).

Many studies have also shown an increased risk of all-cause mortality in patients with increased levels of serum phosphate. Due to this, regular and comprehensive evaluation for CKD-MBD should include serum calcium (Ca), serum phosphate (Phos), serum parathyroid hormone (PTH), serum alkaline phosphatase activity (AlkPhos), and serum 25-hydroxyvitamin D levels (25(OH)D). Patients with CKD stage 5 should also be evaluated for fracture risk, sometimes including bone mineral density testing assessing for osteoporosis. Routine evaluation for CKD-MBD should begin at CKD stage 2. For patients with CKD stage 3-5, frequency of evaluation should be based on rate of progression of CKD, magnitude of abnormality, and baseline levels. More frequent monitoring may be required to assess treatment efficacy and side effects. Serum 25(OH)D < 30 ng/mL should be supplemented with ergocalciferol (Vitamin D2) or cholecalciferol (Vitamin D3).

## 6 End stage kidney disease and renal transplant after HCT

The prevalence of ESKD in adult HCT patients is as high as 4% of patients with CKD (64). Pediatric HCT recipients are unique compared to other ESKD patients given their prior chemotherapy, irradiation, and immunosuppression, as well as their degree of medical complexity with possible pre-existing GVHD, cardiovascular disease, bone disease, diabetes mellitus, short stature, gonadal failure, and risk of opportunistic infections. These patients are also at an increased risk of recurrence of their primary malignancy as well as development of a secondary malignancy, so they will require vigilant surveillance of multiple organ systems. Prolonged immunosuppression not only increases infectious risk in an already immunosuppressed HCT patient, but also can increase the risk of leukemic relapse (53). Due to these challenges, thoughtful multidisciplinary discussion about the risks and benefits of renal transplantation should take place.

Prior to renal transplantation, evaluation for pre-existing immune system impairment should take place when deciding on immunosuppressive agents following renal transplantation. The source of HCT (autologous compared to allogenic) and renal transplant (same allogenic donor compared to a different allogenic donor) can be thought of as biologically distinct groups, especially considering the differences in conditioning regimen (53). Given the presence of “passenger lymphocytes” in solid organ transplants, these donor-derived white blood cells and antigen presenting cells are believed to result in microchimerism which could be both tolerogenic and immunogenic. This can potentially increase the risk of GVHD but also theoretically offer immunologic tolerance in the long term, thereby reducing required immunosuppression (53, 86). Kidney transplant from the same donor can be successful in a patient after HCT without immunosuppression and has been confirmed in multiple studies (53, 87).

Adult data suggests favorable outcomes after renal transplantation in patients who have undergone HCT (53, 88, 89). Hamawi et al. described 10 patients with HCT nephropathy who underwent renal transplant including 6 patients with the same donor who did not receive immunosuppression and 4 patients with different donors (2 living, 2 deceased donors) who did receive immunosuppression. The median estimated graft survival was 105 mo, and there were no episodes of renal transplant rejection. All graft losses (n=4) were due to patient deaths with 3 deaths from an infectious process (2 were not on immunosuppression) and 1 death from myocardial infarction and post-transplant lymphoproliferative disorder (88). Butcher et al. described 6 patients who underwent renal transplant including 3 patients with the same donor who did not receive immunosuppression. Patients were followed up to 31mo and had only 1 mortality from a patient receiving immunosuppression who died from metastatic squamous cell cancer of genital tract (90).

Pediatric literature is limited, but Thomas et al. discusses their single center experience with 3 pediatric patients and Bunin et al. describe 2 pediatric patients (**Table 4**) (53, 91).

**Table 4:** Summary of pediatric literature on renal transplantation post-HCT.

	Age at HCT	Time to renal transplant	Transplant type	Underlying oncologic and renal diagnosis	Current graft survival	Post-renal transplant complications
53	2y	10y	LRKT (pre-emptive)	-Stage III neuroblastoma -Interstitial fibrosis	6y	None
	7y	1y	LRKT (hemodialysis prior)	-Schimke’s immune-osseous dysplasia -Mesangioproliferative glomerulonephritis	3y 8m	None
	4y	10y	LRKT (pre-emptive)	-Stage IV neuroblastoma -Interstitial fibrosis	7m	Tacrolimus toxicity
91	15y	32m	LRKT (pre-emptive)	-Erythropoietic porphyria -CKD following 2 HCTs and prolonged foscarnet for CMV reactivation	1.7y	None
	6y	28m	DDKT (hemodialysis prior)	-Neuroblastoma -TA-TMA requiring bilateral nephrectomies	1.5y	CKD

Living related kidney transplant (LRKT) and deceased donor kidney transplant (DDKT). Pre-emptive refers to patients who were transplanted before starting dialysis.

## 7 Conclusion

Patients undergoing hematopoietic stem cell transplant have unique risk factors for AKI including frequent nephrotoxic exposure, sinusoidal obstruction syndrome, graft-versus-host-disease, and transplant-associated thrombotic microangiopathy, and viral infections such as BK viremia. These patients are at risk for progression to CKD and ESKD. Renal transplantation after HCT has had favorable outcomes in adults with limited pediatric data.

## 8 Future perspectives

Pediatric patients post-HCT are a growing group of high-risk patients who may progress to CKD, ESKD, and potentially renal transplantation in their lifetime. Future studies should focus on better characterizing renoprotective strategies and timely interventions within this population that are distinct from patients with CKD who have not undergone HCT.

## Author contributions

The corresponding author is responsible for ensuring that the descriptions are accurate and agreed upon by all authors. The authors have contributed in multiple roles. KV is responsible for writing the original draft and literature search. JA and CJ are responsible for literature search and editing for the original draft. All authors contributed to the article and approved the submitted version.
